# Study on timing sequence control fracture blasting excavation of deep rock masses with filled joints

**DOI:** 10.1038/s41598-021-00438-9

**Published:** 2021-10-26

**Authors:** Junhong Huang, Guang Zhang, Yi Luo, Xinping Li, Kaiwen Song, Tingting Liu

**Affiliations:** 1 School of Safety Science and Emergency Management, Wuhan University of Technology, Wuhan, 430070 China; 2 Hubei Key Laboratory of Roadway Bridge and Structure Engineering, Wuhan University of Technology, Wuhan, 430070 China; 3 Sanya Science and Education Innovation Park, Wuhan University of Technology, Sanya, China

**Keywords:** Civil engineering, Solid Earth sciences

## Abstract

During the blasting excavation of deep underground caverns, the effects of the structural surface on crack propagation are usually considered in addition to the clamping effects of high in situ stress. Based on the notched borehole and timing sequence control (TSC) fracture blasting method, this paper studies the effects of different borehole shapes on the degree of damage of the surrounding rock and profile flatness of the rock anchor beams and the effects of different filled joint characteristics on the blasting crack propagation rules. The results show that the damage depth of the surrounding rocks by round hole smooth blasting is approximately twice that by notched hole smooth blasting, by which the profile formed is flatter. The notched primary borehole (PBH) remains a strong guidance for crack propagation in a rock mass with filled joints, while the stress concentration effects of the round target borehole (TBH) cannot fully guide the cracks until they fall within a certain distance between the PBH and TBH. It is favourable for cracks to propagate along the lines between boreholes with larger filled joint strengths and larger angles between boreholes.

## Introduction

With the increasing burial depth of underground projects, such as water conservancy, hydropower, mining, national defence construction, and nuclear waste disposal, the clamping effects of the in situ stress on the rock mass must be considered in the blasting excavation of deep underground caverns^1^. Most underground rock masses have complex structural surfaces, such as joints, fractures or bedding fault zones, which usually change the transmission route of the blasting stress wave^2–5^, aggravate the degree of damage of the surrounding rocks, and cause overexcavation and underexcavation of the excavated profile^6,7^. In important parts of underground caverns, such as the rock anchor beam structure, the strength and profile flatness of the surrounding rocks must be controlled to be safe and guarantee the function of the overhead crane on the rock anchor beams.

Most scholars reduce the damage depth, overexcavation and underexcavation by optimizing the borehole shape and initiation circuit. Among them, a popular practice is to change the borehole shape to add a notching process in round boreholes^8^. Zhao^9^ considered notch blasting to be important in developing a free surface for subsequent blasting and affect the overall blasting procedure. Wan^10^ proposed a new specimen of a rectangle plate with a crack and edge notches (RPCEN) to study the fracture toughness of mode-I cracks under a blast load. A notch hole can supply sufficient space to enable the fragments to swell for rock fragmentation^11–13^. Xie^14^ numerically simulated the process of notch blasting under high in situ stresses and the Riedel–Hiermaier–Thoma (RHT) model in LS-DYNA, and they proposed a modified notch blasting design method for deep rock masses. Liang^15^ discovered that the notch tip would suffer from obvious dynamic stress concentration effects at the blasting load in boreholes. Yang^16^ analysed the dynamic propagation behaviours of cracks between boreholes after two notched holes were simultaneously initiated. Jeong^17^ considered that notched blasting helps reduced the degree of damage, overexcavation and underexcavation of tunnel surrounding rocks.

**Figure 1:**
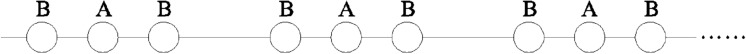
TSC fracture blasting circuit (A refers to PBH and B refers to TBH).

Previously, the main blasting circuit optimization method was to adjust the borehole distance and charging structure. Li^18^ proposed a TSC fracture blasting method, where adjacent boreholes were divided into PBHs and TBHs (Fig. 1). The TBH works as an empty hole before initiation and greatly guides the stress wave^19,20^, which can effectively control the crack propagation^21^. Liu^22^ used the empty hole as a swelling space and surface to study the effect of the distance of the hole to the crack propagation. Cho^23^ studied the guidance of empty holes on cracks via delayed borehole initiation in the model experiment. Yi^24^ studied the superimposed effects of stress waves under a delayed initiation of adjacent boreholes. Through the laboratory delayed borehole initiation test, Johansson^25^ analysed the mutual interaction form and stress distribution of stress waves between PBHs and TBHs and proposed the calculation method of the optimal delayed initiation. Khandelwal^26^ discussed the advantages of the delayed control blasting technology over the surrounding rock vibration reduction through tests.

Although the TSC fracture blasting method can improve the blasting excavation efficiency and cracking results, it is not favourable for round boreholes when attempting to form a good excavation profile in a rock mass with joints, while notched boreholes can greatly improve the profile forming results of a jointed rock mass^27,28^. This paper studies the blasting excavation of a rock mass with filled joints at the rock anchor beam in the deep underground caverns of the Baihetan Hydropower Station based on the TSC fracture blasting method for a PBH notch, to examine the propagation behaviour of cracks in a rock mass with filled joints.

## Field test

### Project situation

Located on the boundary between Sichuan Province and Yunnan Province on the lower reach of the Jinsha River in China, the Baihetan Hydropower Station has a 430,300 km^2^ controlled basin area and a 20.6 billion m^3^ corresponding reservoir capacity. It has 16 units installed in the underground powerhouse, has a 16 million kW initial installed capacity, and an average generating capacity of 60.24 billion kW·h per year. After completion, it will have the largest underground cavern group for any hydropower station in the world, including the main and auxiliary power houses of monocline rock layers, which intersect with the powerhouse axis at an angle of 60° ~ 70° and are dominated by basalt.

The excavation of the rock anchor beam in the underground powerhouse of the Baihetan Hydropower Station is important for the hydropower works of deep underground caverns. It requires controlling the degree of damage of the rock mass and guarantees the excavated profile flatness. The excavation quality of the rock anchor beam directly affects the running safety of the overhead cranes with substantial excavation difficulty and high-quality requirements. Therefore, before the excavation of the rock anchor beam, the blasting excavation test will be performed to propose a proper excavation method and a charging structure.

### Acoustic wave test of the damage depth of surrounding rocks

**Figure 2:**
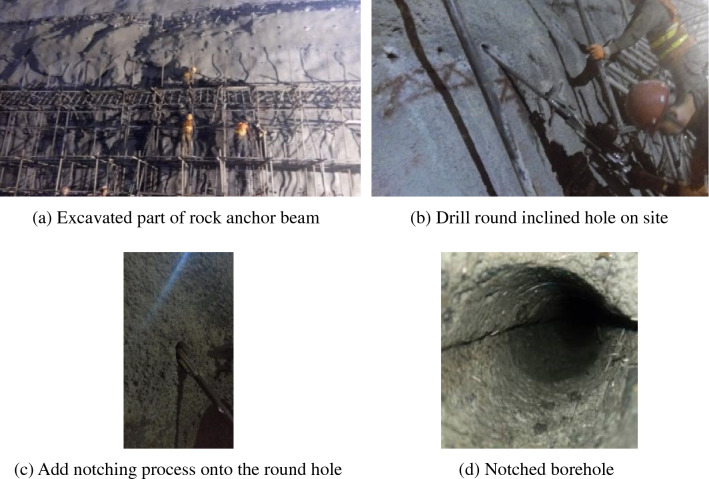
Notched borehole drilling process at rock anchor beam on site.

When a blasting excavation test is conducted for the rock anchor beam, both round and V-notched smooth blasting boreholes are adopted. The notched borehole is the notching process added onto the common round borehole^29^. The borehole notching process on site is shown in Fig. 2, where the top half of Fig. 2a shows the blasting excavation test of the rock anchor beam. First, the round smooth blasting borehole (Fig. 2b) is drilled at the designed part. Second, the notching process is added onto the round hole (Fig. 2c). Finally, the notched borehole shown in (Fig. 2d) is formed. On the right bank of the Baihetan Hydropower Station, three blasting excavation tests have been conducted for the underground main powerhouse, and the borehole charging parameters are shown in Table 1.

**Table 1:** Different borehole layouts and charging parameters.

Borehole name	Borehole diameter/mm	Borehole distance /cm	Borehole depth /cm	Number of borehole	Cartrige diameter/mm	Linear charging density/(g/m)
① Vertical smooth blasting borehole	φ 42	30	248	68	φ 25	65/70/85
② Inclined smooth blasting borehole	φ 42	30	280	68	φ 25	65/70/85
③ Auxiliary borehole	φ 42	90	242	22	φ 25	186/206/250

**Figure 3:**
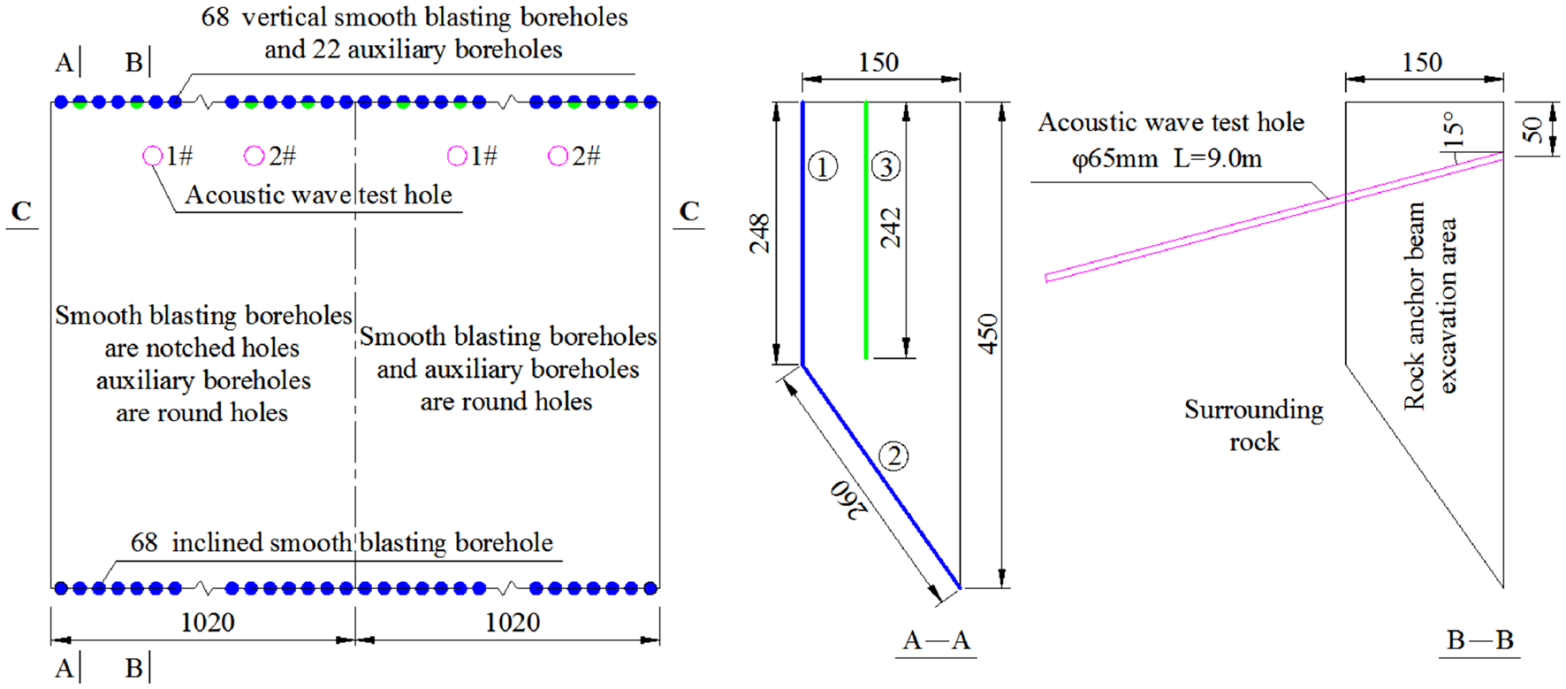
Borehole initiation circuit plan and borehole profile (unit: cm).

**Figure 4:**
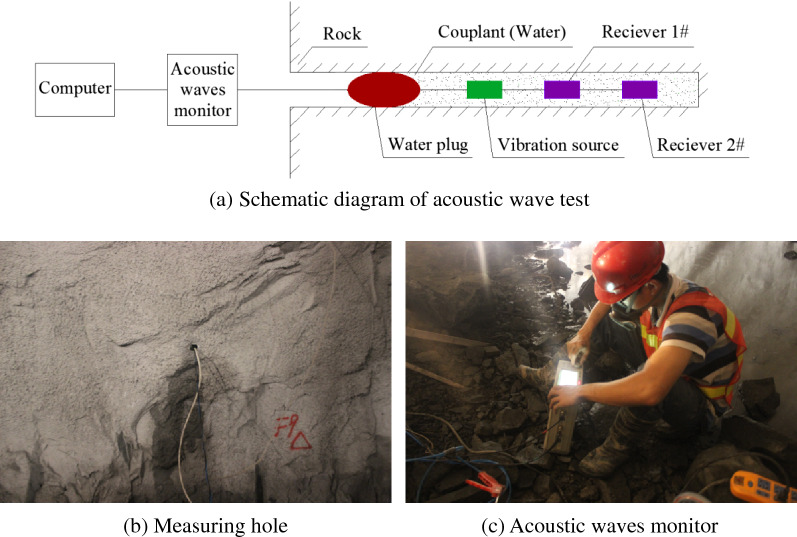
Acoustic wave test of loosening surrounding rock zone.

Between adjacent auxiliary boreholes and between adjacent smooth blasting boreholes, an electric detonator is used for the millisecond delay initiation, while the initiation is in stages between the adjacent auxiliary borehole and the smooth blasting borehole. The borehole initiation circuit plan and borehole profile are shown in Fig. 3, where the smooth blasting boreholes include vertical smooth blasting boreholes and inclined smooth blasting boreholes. Through a comparison between acoustic wave test data and the excavated profile on site, the smooth blasting excavation scheme of the rock anchor beam is discussed to reduce the degree of damage of the surrounding rocks and improve the excavated profile flatness. To analyse the surrounding rock damage conditions due to different borehole shapes, each part of the test is provided with 4 acoustic wave test holes (2 in the notched borehole area and 2 in the round borehole area), which had a depth of 9 m and a diameter of φ65 mm at a slight downward angle to guarantee the coupling effects of water inside the test holes. The basic acoustic wave test method of the loosening surrounding rock zone is shown in Fig. 4. The revelant study participants who appear in these images have obtained their informed consent. All of them allow using their identifying information in this paper. The acoustic wave holes are tested before and after the blasting excavation test to determine the effects of the borehole shape on the damage depth of the surrounding rocks. During the acoustic wave test process on site, the instrument probe is stretched into the lowest point of the bottom of the acoustic wave hole for a P-wave test of the rock mass; then, the instrument is extracted once every 0.2 m for the test. When the reduced wave speed of the two adjacent test points exceeds 10%^30^, the surrounding rock is damaged.

**Figure 5:**
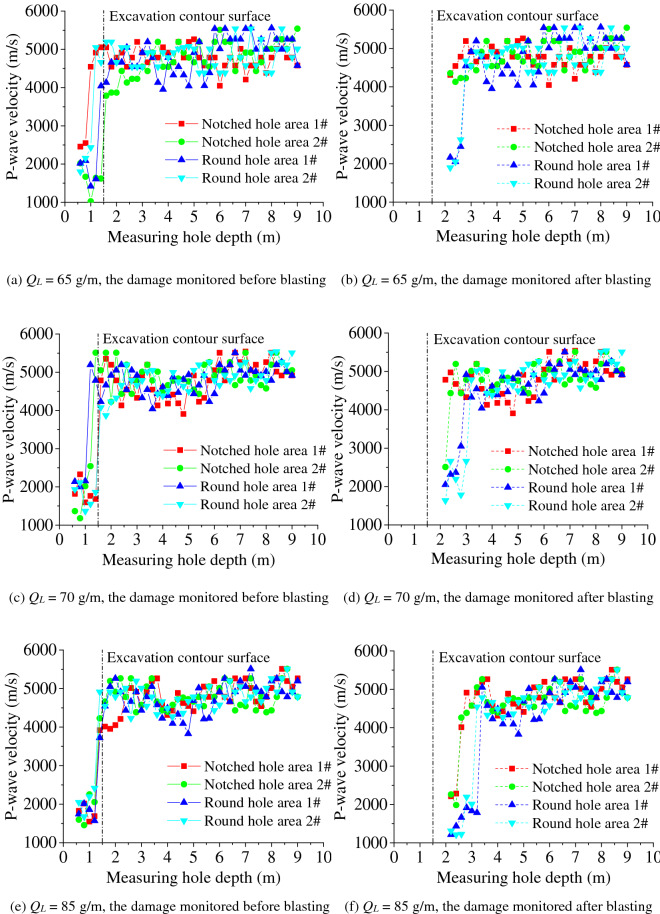
Acoustic wave test data at different linear charging densities before and after blasting.

The acoustic wave test results at different linear charging densities for the boreholes are shown in Fig. 5. In Fig. 5a, c, e, before the rock mass blasting excavation, the acoustic wave speed of the rock mass in the excavated area at 0 ~ 1.4 m above the free face fluctuates at approximately 2,000 m/s, while that of the deep rock mass exceeds 4,000 m/s. Thus, although the rock mass suffers from different degrees of damage under the previous blasting dynamics, its degree of damage essentially does not affect the blasting excavation test of the rock anchor beam. After the blasting excavation, as shown in Fig. 5b, d, f, if the free face before blasting excavation is taken as the benchmark to calculate the damage depth of the surrounding rocks, the surrounding rock in the blasting excavation area with different borehole shapes still suffers from slight damage; when the smooth blasting hole is a round borehole, the damage depth to the surrounding rock is twice as deep as that when the smooth blasting hole is a notched borehole. Thus, the notched blasting hole has a smaller damage depth on the surrounding rock, which can play a role in protecting the surrounding rock.

### Analysis of the excavated profile flatness

**Figure 6:**
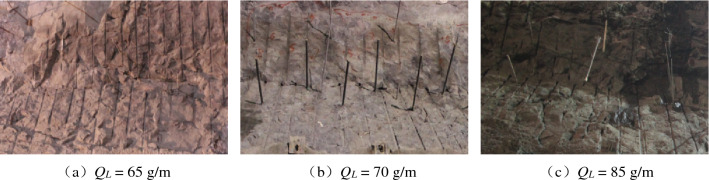
Profile at different linear charging densities by notched borehole blasting excavation.

**Figure 7:**
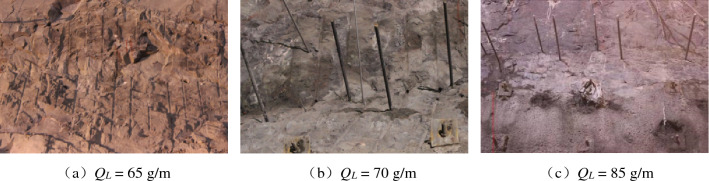
Profile at different linear charging densities by round borehole blasting excavation.

The excavated profile flatness at different linear charging densities is shown in Figs. 6 and 7. The profile formed by the notched borehole blasting excavation is essentially flat, while the excavated profile formed by the round borehole blasting excavation has obvious overexcavation and underexcavation. Thus, notched boreholes greatly help to excavate a smooth and flat profile.

## Numerical calculation model

**Figure 8:**
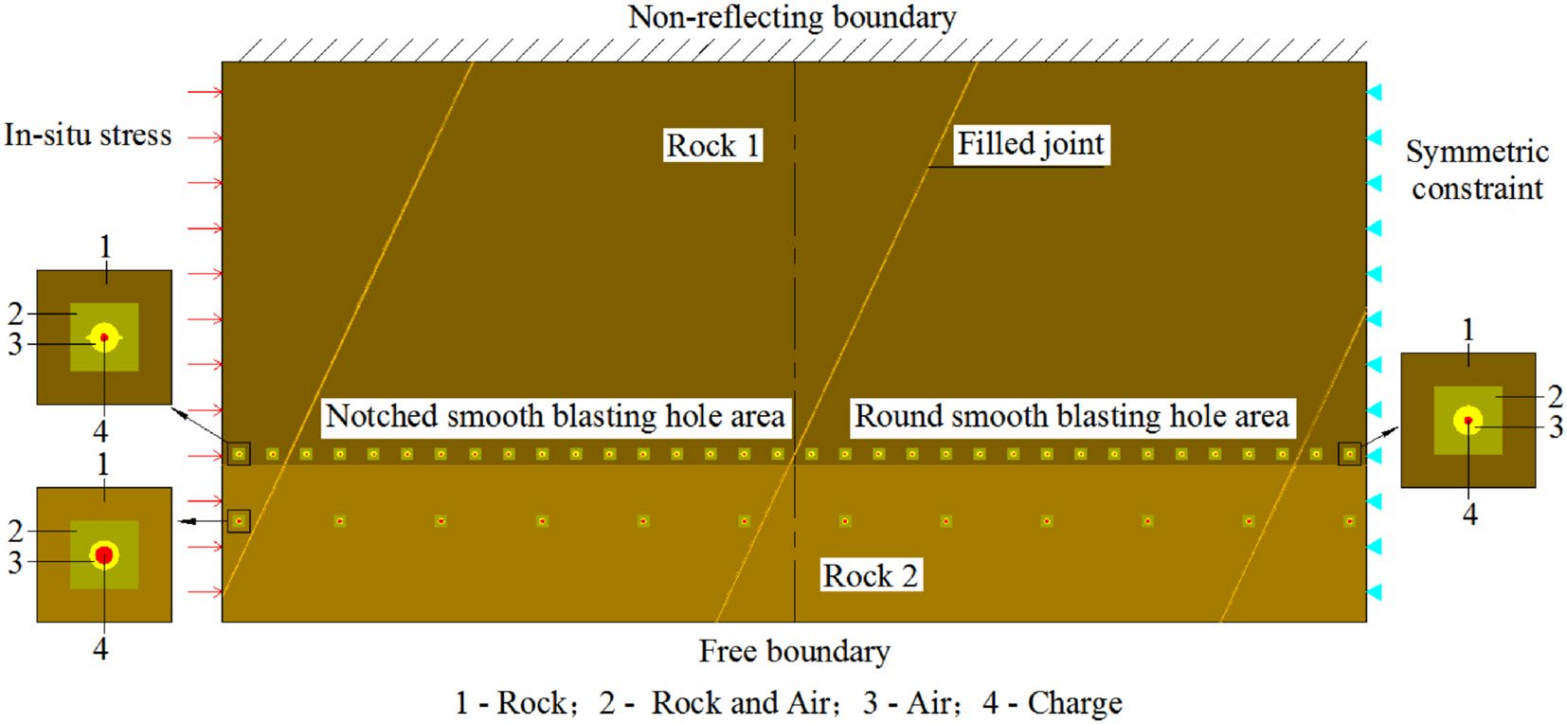
Schematic diagram of 2D numerical model.

Although there are many researchers currently using linear elastic fracture mechanics (LEFM) and acoustic emission to predict fracture propagation in various rock^31–36^, numerical simulation is still a more intuitive and effective method to study the effect of blasting excavation contouring in large underground caverns. A pseudo 3D numerical model is built based on the C–C Profile in Fig. 3. The vertical plan of a vertical smooth blasting borehole is studied, and the calculated area is 10.2 m × 5 m. The model consists of the charge, air, rock mass, and filled joints. The nodes in the innermost layer of rock and those in the outermost layer of air are completely overlapped, so the remaining blasting energy is fully transmitted to the rock layer through the air layer nodes after the energy dissipation to compress the air and other dissipation. The cartridge diameter of the smooth blasting hole and auxiliary hole is calculated per the charge amount with linear charging densities of 70 g/m and 206 g/m, respectively, for equivalent calculations. The top of the model has a non-reflecting boundary, the bottom has a free boundary, the left is applied with in situ stress, the right is applied with fixed constraints, and the thickness direction is applied with vertical symmetric constraints. Although the blasting area is distributed with the joint layer, workers grout the blasting excavation parts in advance to improve the entire continuity of the rock mass structure and reduce the interference of the joint surface on the stress wave transmission and crack propagation. Based on the site conditions, it is determined that the average distance is approximately 4 m, and the inclination is 65° for one set of filled joints of the model. The calculation model is shown in Fig. 8.

### Charge and air state equation

The LS-DYNA program can directly simulate the explosion process of high-energy charges. After the initiation, the volume of the charges is expanded to transmit the pressure produced to the surrounding media. The JWL state equation is used to describe the detonation products^37^, and its equation is

1 $$P = A\left( {1 - \frac{w}{{R_{1} V}}} \right)e^{{ - R_{1} V}} + B\left( {1 - \frac{w}{{R_{2} V}}} \right)e^{{ - R_{2} V}} + \frac{{wE_{1} }}{V}$$

where *V* is the relative volume of the detonation gas, *E*_1_ is internal energy, and *A*, *B*, *R*_1_, *R*_2_, and *w* are the material parameters of the state equation.

For the Baihetan underground works, the 2# rock emulsion explosive and JWL state equation parameters are shown in Table 2.

**Table 2:** Basic charge parameters and the JWL state equation parameters.

*ρ*_1_/(g/cm^3^)	*D*/(m/s)	*A*/GPa	*B*/GPa	*R*_1_	*R*_2_	*w*
1.0	3200	214	0.18	4.15	0.95	0.13

*ρ*_1_ is the charge density and *D* is the detonation wave speed.

The state equation of the air media model is expressed by an equation of State (EOS) linear polynomial^38^. The specific parameter values are shown in Table 3.

2 $$P = C_{0} + C_{1} \mu + C_{2} \mu^{2} + C_{3} \mu^{3} + (C_{4} + C_{5} \mu + C_{6} \mu^{2} )E$$

where *C*_0_, *C*_1_, *C*_2_, *C*_3_, *C*_4_, *C*_5_, and *C*_6_ are the 1st, 2nd, 3rd, 4th, 5th, and 6th polynomial equation coefficients, respectively; *C*_2_*μ*^2^ and *C*_6_*μ*^2^ are set to zero if *μ* < 0, *μ* = *ρ/ρ*_0_ − 1; *ρ/ρ*_0_ is the ratio of the current density to the reference density, and *ρ*_0_ is a defined nominal or reference density.

**Table 3:** EOS linear polynomial parameters.

*C*_0_	*C*_1_	*C*_2_	*C*_3_	*C*_4_	*C*_5_	*C*_6_	*E*_0_/GPa	*V*_0_
0	0	0	0	0.4	0.4	0	2.5e−06	1.0

*E*_0_ is the initial internal energy per unit reference for a specific volume, and *V*_0_ is the initial relative volume.

### Calculation model of rocks and filled joints

When the charge is exploded, the rock strain in near areas is very large, there are obvious strain rate effects, and the plastic hardening model with strain rate effects is used.

3 $$\sigma_{y} = \left[ {1 + \left( {\frac{{\dot{\varepsilon }}}{C}} \right)^{\frac{1}{P}} } \right]\left( {\sigma_{0} + \beta E_{p} \varepsilon_{p}^{eff} } \right)$$

4 $$E_{p} = \frac{{E_{0} E_{\tan } }}{{E_{0} - E_{\tan } }}$$

where $\sigma_{0}$ is the initial yield stress of rock; *E*_0_ is the elastic modulus; $\dot{\varepsilon }$ is the loading strain rate; *C* and *P* are taken as 2.5/s and 4.0, respectively^39^; *E*_p_ is the plastic hardening modulus of rock mass; *E*_tan_ is the tangent modulus; $\beta$ is the hardening parameter of the isotropic hardening and kinematic hardening construction (0 ≤ $\beta$ ≤ 1); and $\varepsilon_{p}^{eff}$ is the effective plastic strain of the rock mass, which is defined below:

5 $$\varepsilon_{p}^{eff} = \int_{0}^{{t_{a} }} {d\varepsilon_{p}^{eff} }$$

6 $$d\varepsilon_{p}^{eff} = \sqrt {\frac{2}{3}d\varepsilon_{ij}^{p} d\varepsilon_{ij}^{p} }$$

where $t_{a}$ is the accumulated time of the plastic strain, and $\varepsilon_{ij}^{p}$ is the component of the plastic strain deviation of the rock mass.

The principle of rock mass damage depends on the property of rock mass as well as the practical force conditions. The pressure of rock mass, taking the Mises damage rule, forms the crushing area of rock mass blasting, while the cracks area is the result of the damage of tensile force. The damage rule of rock mass is as follows:

7 $$\left. {\begin{array}{*{20}l} {\sigma_{VM} > \sigma_{cd} \left( {{\text{crushs}}\,{\text{area}}} \right)} \hfill \\ {\sigma_{t} > \sigma_{td} \left( {{\text{cracks}}\,{\text{area}}} \right)} \hfill \\ \end{array} } \right\}$$

8 $$\sigma_{VM} = \sqrt {\frac{3}{2}\sigma_{ij} \sigma_{ij} }$$

where, $\sigma_{VM}$ is the von Mises effective stress of any point in rock mass; $\sigma_{ij}$ (*i, j* = 1, 2, 3) is the stress components of rock mass. $\sigma_{t}$ is the tensile stress of explosion load of any point in rock mass; $\sigma_{cd}$, $\sigma_{td}$ are known as uniaxial dynamic compressive strength and tensile strength of rock mass respectively.

The dynamic compressive stress of rock increases with the improvement of loaded strain rate, generally approximated by the following equation^40^:

9 $$\sigma_{cd} = \sigma_{c} \dot{\varepsilon }^{\frac{1}{3}}$$

where, $\sigma_{c}$ in the equation refers to the uniaxial static compressive stress of rock.

The loaded strain rate of rock $\dot{\varepsilon }$ in blasting during programs is within 10^0^–10^5^ s^−1^, among which the strain rate in crushed zones could be $\dot{\varepsilon } = 10^{2} { - }10^{4} {\text{s}}^{{{ - }1}}$ and in cracked zones could be $\dot{\varepsilon } = 10^{0} { - }10^{3} {\text{s}}^{{{ - }1}}$.

For the lack of corresponding analytical data of experiments and theories, the value of dynamic tensile strength approximates:

10 $$\sigma_{td} = \sigma_{t} \dot{\varepsilon }^{\frac{1}{3}}$$

where $\sigma_{t}$ in the equation refers to the uniaxial static tensile strength of rock mass.

When the maximum tensile stress (*σ*_*td*_) of a dangerous point in the rock mass reaches its extreme tensile strength, damage will occur. To ensure the accuracy of the results, a proper method to get the mechanical properties of the rock mass is very important^41–44^. Asem and Gardoni^45^ presented a generalized Bayesian approach to develop probabilistic predictive models for the rock mass properties. A new procedure for the design of drilled piers socketed into soft rock is presented and the selection of design parameters discussed by Rowe and Armitage^46^. Hoek and Diederichs^47^ Based on data from a large number of in situ measurements from China and Taiwan a new relationship, based upon a sigmoid function, is proposed. The properties of the intact rock as well as the effects of disturbance due to blast damage and/or stress relaxation are also included in this new relationship. According to the above analysis, combined with the experimental research conducted by drilling cores from different parts of the surrounding rock of Baihetan Hydropower underground powerhouse, the parameters used in the numerical simulation of the rock and the filling joint material were selected as shown in Table 4.

**Table 4:** Physical and mechanical parameters of the rock and filled joints for calculation.

Material	Density *ρ*/(g/cm^3^)	Elastic modulus *E*_0_/GPa	Poisson's ratio *µ*	Tangent modulus *E*_*tan*_/GPa	Uniaxial tensile strength *σ*_*t*_/MPa	Uniaxial compressive strength *σ*_*c*_/MPa	Hardening parameter *β*
Rock 1	2.7	50.0	0.25	18	6.0	70.0	1
Rock 2	2.5	45.0	0.26	16	5.0	60.0	1
Filled joint	1.8	25.0	0.29	10	3.0	35.0	1

### Analysis of the calculation results

**Figure 9:**
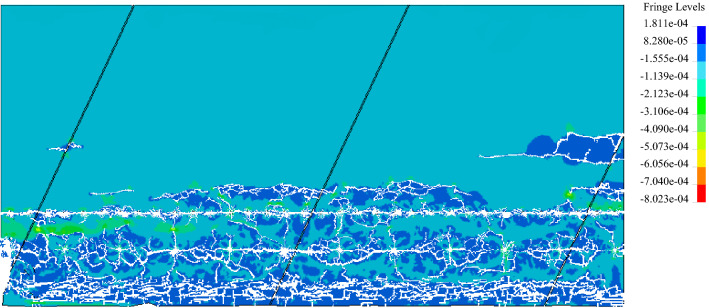
Crack propagation of blasting excavation of rock mass model within 5 ms (positive values represent tensile stresses and negative values represent compressive stresses).

When the previous geological conditions of the rock mass are similar, the positive value in Fig. 9 indicates that the rock mass is under tension, and the negative value indicates that the rock mass is under compression. When the dynamic tensile stress of some rock mass elements reaches its dynamic tensile strength in the calculation process, these rock mass elements will disappear and show the process of crack formation. The 5 ms model calculation shows that most of the damage depth of the surrounding rocks in notched smooth blasting holes is 0.5 ~ 0.6 m, and in round smooth blasting holes, it is 0.6 ~ 1.3 m, which is similar to that of the excavation of the rock anchor beam on site. Generally, the degree of damage in a round borehole area is approximately twice that in a notched borehole area, and the smooth blasting surface in the notched hole area is slightly flatter than that in the round hole area. Thus, the model can better simulate the blasting excavation process of the rock mass with filled joints in practical projects.

## Numerical analysis of the TSC fracture blasting method for a PBH notch

**Figure 10:**
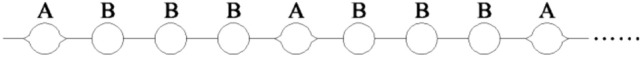
TSC fracture blasting circuit for PBH notch.

The above research describes that the notched hole also well guides detonation cracks in rock masses with filled joints. Since it takes approximately twice as long to drill a notched hole than a round hole, the construction process of blast excavating the underground powerhouse will be affected. Based on the TSC fracture blasting excavation method proposed by Li^48^, only PBHs are notched in the rock mass model with filled joints; the distance between adjacent boreholes is taken as approximately 19 times the diameter of the boreholes, i.e., 800 mm; the delayed initiation time between PBH and TBH is taken as 1 ms. To compare and analyse the effects of the round target borehole with and without stress concentration effects on the crack propagation, the method in Fig. 10 is used to connect the smooth blasting borehole circuit.

**Figure 11:**
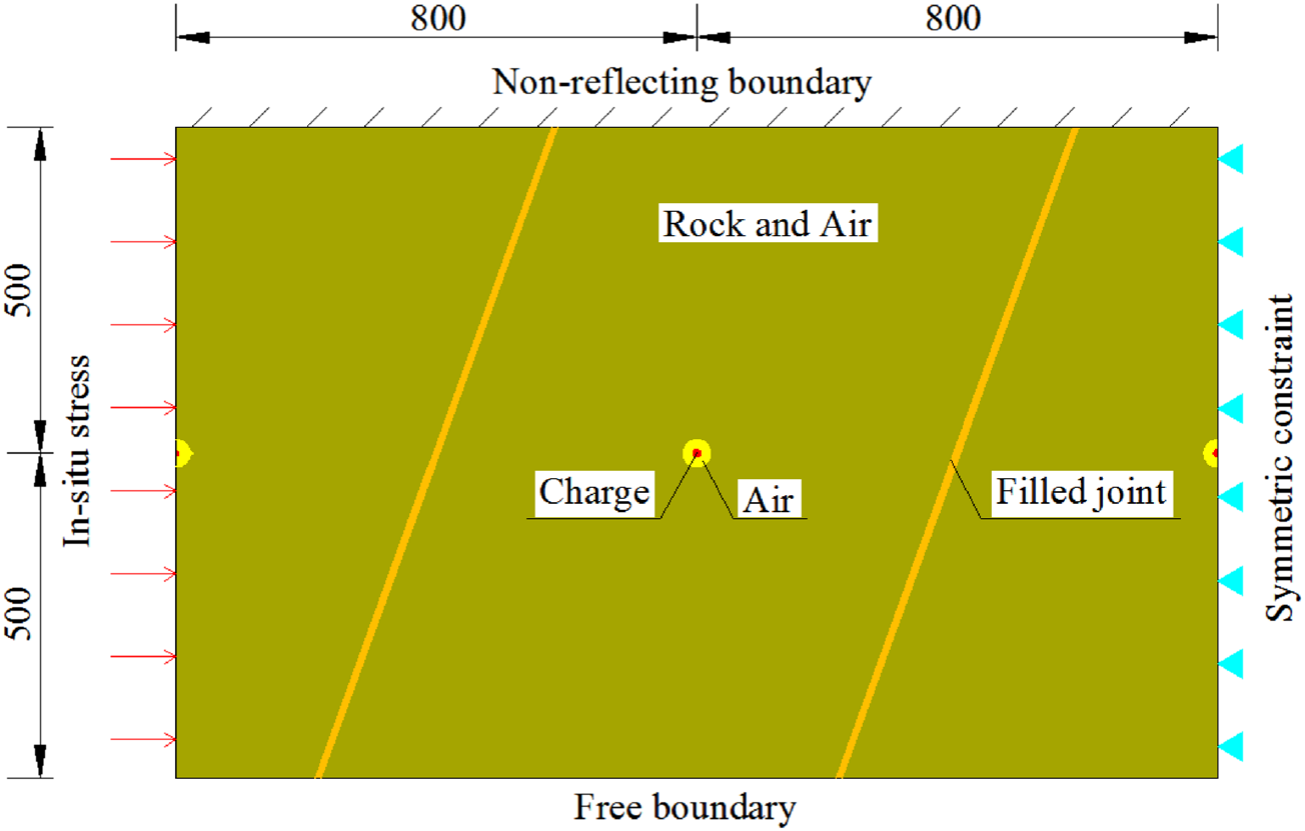
Schematic diagram of TSC fracture blasting numerical model with notched PBH at different joint directions (unit: mm).

As shown in Fig. 11, the built symmetric model consists of three boreholes: the notched PBH on the left and round TBHs in the middle and on the right. The angles of the joints with a line between boreholes are 15°, 30°, 45°, 60°, 75°, and 90°. Based on the physical and mechanical property change rules of rock, the parameters of the four filled joints are shown in Table 5.

**Table 5:** Physical and mechanical parameters of the rock and filled joints.

Material	Density *ρ*/(g/cm^3^)	Elastic modulus *E*_0_/GPa	Poisson's ratio *µ*	Tangent modulus *E*_*tan*_/GPa	Uniaxial tensile strength *σ*_*t*_/MPa	Uniaxial compressive strength *σ*_*c*_/MPa	Hardening parameter *β*
Rock	2.7	50.0	0.25	18.0	6.0	70.0	1
Filled joint 1	1.2	10.0	0.32	4.0	0.5	5.0	1
Filled joint 2	1.4	15.0	0.31	6.0	1.0	15.0	1
Filled joint 3	1.6	20.0	0.30	8.0	2.0	25.0	1
Filled joint 4	1.8	25.0	0.29	10.0	3.0	35.0	1

**Figure 12:**
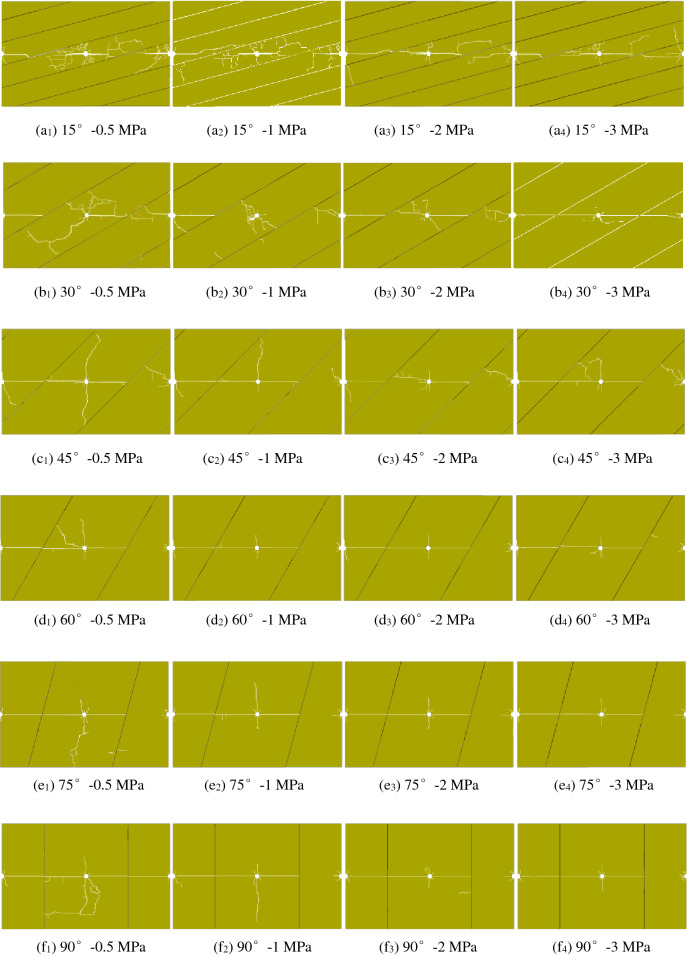
Crack penetrating results at different joint directions and tensile strengths.

Based on the in situ stress of the rock anchor beam of the underground powerhouse at the Baihetan Hydropower Station, the in situ stress is approximately taken as 20 MPa. The crack propagation through the results of rock mass models with different filled joint characteristics and directions are shown in Fig. 12.

As shown in Fig. 12, in the rock mass with filled joints, even if there are reflection effects of the joints on the stress wave, the cracks produced from notched PBHs mainly propagate along the line between the boreholes and the rock mass model, and a filled joint layer shows a better guidance of notched holes on the cracks. After two round TBHs are simultaneously initiated, the round borehole in the middle suffers from obvious stress concentration effects due to the blasting stress wave of the PBH, most of the cracks after initiation are propagated along the line between the boreholes, and several secondary cracks vertically propagate to the joint surface only when the joint angle is small. Since the round borehole on the right is free of obvious stress concentration effects before initiation, at a joint angle of 15° ~ 45°, the borehole is near the joint surface and suffers from a strong reflection stress wave at the joint surface, and the produced cracks mainly vertically propagate to the joint surface. At a joint angle of 60° ~ 90°, it is difficult for the blasting pressure and stress wave reflecting force in the borehole to facilitate the propagation of the cracks.

With identical joint angle, when the filled joint strength is 0.5 MPa and 1 MPa, there are many secondary cracks near the TBHs. When the filled joint strength is 2 MPa and 3 MPa, the joint surface has a reduced reflecting force on the blasting stress wave, and the number of secondary cracks between boreholes is also reduced to reduce the degree of damage of the surrounding rocks and be favourable for forming a flatter excavation profile.

With identical joint strength, when the joint angle is 15° and 30°, severe damaged areas occur near the middle TBH. However, with an increasing joint angle, the reflection effects of the stress wave on the rock mass between adjacent joints gradually decrease, and the secondary cracks near the TBH are accordingly reduced to decrease the degree of damage of the surrounding rocks and help form a flat excavation profile between boreholes.

**Figure 13:**
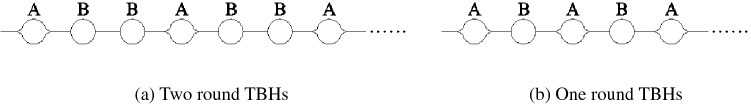
TSC fracture blasting circuit for PBH notch.

To summarize, with a PBH notch, to fully propagate the stress concentration effects of TBHs, the best method is to arrange only one or two round TBHs between PBHs, as shown in Fig. 13. A larger angle and a higher strength of filled joints contribute to substantial penetration and a flat excavation profile between boreholes, and the degree of damage of the surrounding rocks decreases. Based on a strike angle of 60° ~ 70° between the joints on site and the cavern axis, this method decreases the rock mass damage depth and accelerates the rock mass excavation speed. When the filled joint has a small angle and a low strength, all boreholes can be notched, which makes the cracks mainly propagate along the line between the boreholes and reduces the number of propagating secondary cracks, as shown in Fig. 12a, e.

## Conclusions

Based on the acoustic wave test, profile flatness comparison, and the numerical simulation analysis, the following conclusions are made:

1. The damage depth of the surrounding rocks via round borehole smooth blasting is approximately twice that of the notched borehole smooth blasting, and the profile formed by notched borehole blasting excavation is flatter than that of a round borehole.
2. In the TSC fracture blasting excavation method, the TBH with obvious stress concentration effects caused by PHB notching more easily produces penetrating cracks along the line between boreholes. By controlling the number of adjacent TBHs, we can decrease the degree of damage of the surrounding rocks while improving the blasting excavation efficiency.
3. When the strength of the filled joints and angle of joints with a line between boreholes increase, it is more favourable for cracks to propagate along the line between boreholes during the jointed rock mass blasting excavation.

## Discussion

In this paper, the crack propagation law under different hole shapes and detonation timing sequences is mainly based on numerical simulations. But the mechanical properties of rock mass are more complicated in actual engineering. So more advanced test methods are needed to get more accurate mechanical properties of rock masses to verify the numerical simulation results, and optimize the new method proposed in this paper in the underground cavern blasting excavation process.
